# Molecular species of oxidized phospholipids in brain differentiate between learning- and memory impaired and unimpaired aged rats

**DOI:** 10.1007/s00726-022-03183-z

**Published:** 2022-07-11

**Authors:** Marie-Sophie Narzt, Christopher Kremslehner, Bahar Golabi, Ionela-Mariana Nagelreiter, Jovana Malikovic, Ahmed M. Hussein, Roberto Plasenzotti, Volker Korz, Gert Lubec, Florian Gruber, Jana Lubec

**Affiliations:** 1 Department of Dermatology, Medical University of Vienna, Vienna, Austria; 2 Ludwig Boltzmann Institute for Experimental and Clinical Traumatology, AUVA Research Center, Linz/Vienna, Austria; 3 Center for Brain Research, Department of Molecular Neurosciences, Medical University of Vienna, Vienna, Austria; 4 Department of Pharmaceutical Chemistry, Faculty of Life Sciences, University of Vienna, Vienna, Austria; 5 Programme for Proteomics, Paracelsus Private Medical University, Salzburg, Austria; 6 Department of Zoology, Faculty of Science, Al-Azhar University, Assiut, Egypt; 7 Center for Biomedical Research, Division of Laboratory Animal Science and Genetics, Medical University of Vienna, Himberg, Austria

**Keywords:** Prefrontal cortex, Memory, Phospholipids, Learning

## Abstract

Loss of cognitive function is a typical consequence of aging in humans and rodents. The extent of decline in spatial memory performance of rats, assessed by a hole-board test, reaches from unimpaired and comparable to young individuals to severely memory impaired. Recently, proteomics identified peroxiredoxin 6, an enzyme important for detoxification of oxidized phospholipids, as one of several synaptosomal proteins discriminating between aged impaired and aged unimpaired rats. In this study, we investigated several components of the epilipidome (modifications of phospholipids) of the prefrontal cortex of young, aged memory impaired (AI) and aged unimpaired (AU) rats. We observed an age-related increase in phospholipid hydroperoxides and products of phospholipid peroxidation, including reactive aldehydophospholipids. This increase went in hand with cortical lipofuscin autofluorescence. The memory impairment, however, was paralleled by additional specific changes in the aged rat brain epilipidome. There was a profound increase in phosphocholine hydroxides, and a significant decrease in phosphocholine-esterified azelaic acid. As phospholipid-esterified fatty acid hydroxides, and especially those deriving from arachidonic acid are both markers and effectors of inflammation, the findings suggest that in addition to age-related reactive oxygen species (ROS) accumulation, age-related impairment of spatial memory performance has an additional and distinct (neuro-) inflammatory component.

**Supplementary Information:**

The online version contains supplementary material available at 10.1007/s00726-022-03183-z.

## Introduction

Phospholipids (PL) make up the majority of the lipid mass (up to 50%) of the mammalian brain. The major phospholipid classes of the human brain are phosphatidylcholines (PC), phosphatidyl-ethanolamines, and phosphatidylserines, and this pattern is found in all regions of the cerebral cortex. The most frequent among the PCs have a palmitic or stearic acid esterified at the sn-1 position, whereas especially the brain is rich in PL with polyunsaturated fatty acids (PUFA) at the *sn*-2 position. In general, the most abundant PUFA in human brain are docosahexaenoic acid (DHA) and arachidonic acid (AA) (Choi et al. 2018; Jove et al. 2019). The rat (prefrontal) cortex is also rich in these phospholipid classes and similarly rich in esterified PUFA (Miranda et al. 2019). PUFA-containing PL (PUFA-PL) are regarded the most important oxidizable molecules of the brain (Anthonymuthu et al. 2018; Falomir-Lockhart et al. 2019). Oxidation of PUFA moieties can either occur non-enzymatically or be specifically mediated by enzymes. PUFA-PL are essential for integrity of cell membranes and cellular signaling (Bochkov et al. 2010), but upon oxidation, they acquire novel biological functions including the interaction with several cell surface receptors and thereby modulating cell signaling cascades (Spickett and Pitt 2015). Both detrimental and protective biological functions have been ascribed to oxidized lipids, from being cytotoxic or pro-apoptotic agents to being inflammation mediators (Aldrovandi and O'Donnell 2013; Bochkov et al. 2017; Furnkranz and Leitinger 2004). However, oxidized lipids also have important roles in limiting acute inflammation reactions, as they can interfere with Toll-like receptor (TLR) signaling, induce important anti-inflammatory or tolerance pathways (Greig et al. 2012; Serbulea et al. 2017).

Free radicals and non-radical reactive oxygen species (ROS) like singlet oxygen can initiate non-enzymatic oxidation of PUFA-PL, yielding phospholipid hydroperoxides (PL-OOH). The radicals and ROS can derive from defects in the mitochondrial electron transport chain, which are elevated in cellular senescence and tissue aging, from extrinsic stressors, or as products of NADPH oxidase and other enzymes. Elevated levels of PL-OOH are potentially dangerous for cell integrity and survival, as PL-OOH hydroperoxides can initiate a lipid peroxidation chain reaction. These lipid molecular species have been described in age-related conditions including atherosclerotic vessels or in tissue of stroke patients (reviewed in (Bochkov et al. 2017)). Hydroxides of the esterified fatty acids (PL-OH), including PC-esterified hydroxyeicosatetraenoic acids (HETE-PC), derive from the reduction of the PL-OOH or the action lipoxygenases (LOX) on the attached or free polyunsaturated fatty acid (Watson et al. 1997). Esterified HETE-PC and HETE-PE are inducible by pro-inflammatory agonists in mammalian infection (O'Donnell and Murphy 2012).

Several novel roles for phospholipid oxidation are recently being related to aging processes and age-related neurodegeneration, as in Alzheimer's disease (Ademowo et al. 2017, 2020; Dias et al. 2020; Frisardi et al. 2011; Haider et al. 2011). We have recently demonstrated accumulation of specific OxPL (azelaic acid esterified PC) in the brains of mice which had autophagy deleted under the tyrosinase promoter and displayed an age-related neurodegenerative phenotype (Sukseree et al. 2018). Relations to specific learning- and memory processes, however, are yet not shown yet. Alzheimer’s disease and other neurodegenerative disorders are characterized by general memory loss and learning impairment. Lipid metabolism is changed in aged humans and rodents. Kim and colleagues (Kim et al. 2017) found dysregulated metabolism of phosphatidylcholines and ceramides in the plasma of Alzheimer’s disease patients and tandem mass spectrometry (MS/MS) lipidomics are the method of choice to detect subtle changes of the lipidome in brain disease and upon pharmacological or dietary intervention (Hanson et al. 2019). Recently, we found that a number of lipids classes, namely glycerophosphocholines, glycerophosphoethanolamines, hexosyceramides, and sphingomyelins, were downregulated in the hippocampus of young male rats showing an impairment of spatial cognitive flexibility (Malikovic et al. 2019). However, there is evidence that lipid processes in the frontal cortex are also involved the formation and consolidation of spatial reference memories (Moreira et al. 2012; Zamzow et al. 2019), probably based on the connections to the hippocampus. Thus, it is feasible that changes in specific lipid classes may contribute to the often observed loss of spatial learning and memory abilities in aged rats. Therefore, we tested aged rats in the spatial reference learning hole-board task. We identified memory impaired and unimpaired rats and measured PL oxidation products to reveal whether they may serve as bio-indicators of cognitive impairment in aged subjects. Young animals served as controls. We specifically investigated the relative changes in PFC and their bioactive oxidation products. We found that while aging resulted in elevation of phospholipid hydroperoxides, of lysophospholipids, and of aldehydophospholipids in the prefrontal cortex, rats with impaired memory showed additional regulation of other specific oxidized phospholipid species. The aged memory impaired rats showed elevated phospholipid hydroxide levels especially of arachidonic acid-derived species, while azelaoyl phospholipid modifications were decreased in comparison to rats with unimpaired memory. The results show that impaired memory is accompanied by the regulation of specific cortical phospholipid species rather than a mere elevation of the age-associated phospholipid oxidation products. Thus, the lipid species associated with impaired memory may be specific targets for dietary and pharmacological intervention.

## Methods

### Animals

Aged (17–18 months) and young (4 months) male Sprague–Dawley rats that were bred and kept in the Core Unit of Biomedical Research, Division of Laboratory Animal Science and Genetics, Medical University of Vienna were used. Rats were housed in groups of three in standard Makrolon cages filled with autoclaved woodchips (temperature: 22 ± 2 ℃; humidity: 55 ± 5%; 12 h artificial light/12 h dark cycle: light on at 7:00 am). Tap water and food was provided ad libitum. The study was carried out according to the guidelines of the Ethics committee, Medical University of Vienna, and was approved by the Federal Ministry of Education, Science and Culture, Austria.

All subjects were on a low-calorie diet (ssniff, R/M-H Ered II, Soest, Germany) for a period of 4 months (in case of aged animals) or 7 weeks (in case of young animals) prior to behavioral and physiological assessment. The aged animals represent a subgroup of animals from a previous study (Malikovic et al. 2018).

### Hole-board

All groups underwent the hole-board test before samples were taken. A hole-board test was performed as previously described with minor modifications (Smidak et al. 2017). The hole-board board (1 m × 1 m) was made of black plastic surrounded by translucent plexiglass walls equipped with proximal spatial cues. Surrounding room structures served as distal cues. Four out of sixteen regularly arranged holes (diameter and depth 7 cm) were baited (dustless precision pellets, 45 mg, Bioserv^®^, Flemington, NJ; USA) with the same pattern during the entire test. A second board below was covered with food pellets to avoid olfactory orientation. Ten-minute duration handling sessions per day for 4 days prior to the experiment made the rats familiar to the experimenter. Experimenter habituation was followed by 2 days of habituation to the hole-board by free exploration of the maze for 15 min each day with access to food pellets. Controlled food restriction reduced the weight of the rats to 85% of their initial body weight. Tap water was given ad libitum. Animals were trained over 3 days (five trials on day 1, four trials on day 2, and a retention trial at day 3) with an inter-trial interval of 20 min for individual rats. Trial duration was 120 s or until all four pellets were eaten. The apparatus was cleaned with 0.1% Incidin between trials to remove odor cues of individual rats. Performance of the rats was recorded by a video camera and stored on a computer. The hole visits and removals of pellets were noted for each trial. To compare rats with similar levels of motivation, rats with less than 40 hole visits in total over the ten trials were excluded from the analysis (Kristofova et al. 2018).

Reference memory errors were noted as the number of visits to the unbaited holes. Reference Memory Index (RMI) was calculated using the formula (first + revisits of baited holes)/total visits of all holes. All behavioral training/testing was performed during the light phase of the light–dark cycle. The learning index was calculated as the mean value of reference indices of trials 6–9 at day 2. The memory index is represented by the reference memory index of the retention trial 10 (day 3).

As a “impaired” was characterized rats having either learning or memory indices lower than one standard deviation from the mean and “unimpaired” having indices one standard deviation higher than the mean, whereas learning and memory indices had to be higher, respectively, lower than mean values. The rats analyzed in the present study were randomly chosen from unimpaired (19 animals in total) and impaired (15 animals) animals from a larger cohort of rats (*n* = 127).

### Tissue preparation

Using a long microspatula, the brain was placed on a para cooler (RWW Medizintechnik, Germany) and the temperature was kept at 6 ℃. Dissection of the frontal lobe was performed by making a coronal cut exactly at the rostral boundary of the hypothalamus, approximately 2.7–3 mm below the olfactory bulb. The preparation process took 5 min per animal. Tissues were immediately transferred into labeled tubes containing methanol/acetic acid (3%)/butylated hydroxytoluene [BHT, 0.01%w/v, as antioxidant which strongly inhibits oxidation of PC (PMID: 17900550)] and stored at -80 degrees Celsius until tissue was ready for further lipid processing below. For histology, the tissues were embedded in optimal cutting temperature (OCT) compound in cryo-molds and snap-frozen in liquid nitrogen.

### Lipid measurement

Brain tissue of the frontal lobe (*n* = 5 per group) was homogenized in the ninefold volume of methanol/acetic acid (3%)/butylated hydroxytoluene (BHT, as antioxidant, 0.01%). Samples were purified using the liquid–liquid extraction procedure (Gruber et al. 2012) and reconstituted in 85% aqueous methanol containing 5 mM ammonium formate and 0.1% formic acid. Analysis was performed at FTC-Forensic Toxicological Laboratory, Vienna. Samples were separated with a core–shell type C18 column (Kinetex 2.6 μm, 50 mm × 3.0 mm ID; Phenomenex, Torrance, CA) kept at 20 °C and using a 1200 series HPLC system (Agilent, Waldbronn, Germany) in reversed phase. Elution was performed by a linear gradient of water (eluent A) and methanol (eluent B) both containing 5 mM ammonium formate and 0.1% formic acid. The time program was: 0 min: 85% B; 2 min: 85% B; 3 min: 95% B; 11 min: 100% B; 16 min: 100% B; 16.1 min: 85% B; 20 min: 85% B. The flow rate of the mobile phase was 0.4 ml/min from 0 to 3 min and was linearly increased to 0.6 ml/min until 11 min run time, kept constant at 0.6 ml/min until 19 min run time, and was reduced linearly to 0.4 ml/min until 20 min run time.

Detection was performed with a 4000 QTrap triple quadrupole linear ion trap hybrid mass spectrometer system with a Turbo V electrospray ion source (Applied Biosystems, Foster City, CA, USA) in positive-ion mode by selected reaction monitoring (SRM) of 99 MS/MS transitions using product ion (*m/z* 184), the diagnostic fragment for the phosphocholine residue. The electrospray ionization voltage was set to 4500 V, and the temperature of the ion source was set to 550 ℃. Nitrogen was used as nebulizer, heater, and curtain gas with the pressure set at 50, 30, and 20 psi, respectively. SRM settings were as follows: declustering potential = 120, entrance potential = 10, collision energy = 53, cell exit potential = 14, and dwell time = 13 ms.

Data acquisition and instrument control were performed with Analyst software, version 1.6 (Applied Biosystems). Analyte peak areas were normalized to 1,2-di-palmitoyl- sn-glycero-3-phosphorylcholine (DPPC), a saturated species of brain phosphocholine, and multiplied with factor 10.000 to present the relative changes in the oxidized species which are lower than DPPC in numbers larger than one, as in (Gruber et al. 2012), where also external calibration for selected lipid species was described. Non-oxidized native lipid species (1-palmitoyl-2- arachidonoyl-sn-glycero-3-phosphorylcholine (PAPC) *m/z* 782; 1-palmitoyl-2-linoleoyl-sn-glycero-3-phosphorylcholine (PLPC) *m*/*z* 758; 1-stearoyl-2-arachidonoyl-sn-glycero-3-phosphorylcholine (SAPC) *m/z* 810; 1-stearoyl-2-linoleoyl-sn-glycero-3-phosphorylcholine (SLPC) *m/z* 786) and chain fragmented oxidized species (1-palmitoyl-2-(5-oxovaleroyl)-sn-glycero-3-phosphorylcholine (POVPC) *m/z* 594; 1-palmitoyl-2-azelaoyl-sn-glycero-3-phosphorylcholine (PAzPC) m/z 666; 1-stearoyl-2-azelaoyl-sn-glycero-3-phosphorylcholine (SAzPC) *m/z* 694 1-palmitoyl-2-glutaroyl-sn-glycero-3-phosphorylcholine (PGPC) *m/z* 610; 1-palmitoyl-2-(oxo-nonanoyl)-sn-glycero-3-phosphorylcholine (PONPC) *m/z* 650 and 1-stearoyl-2-(oxo-nonanoyl)-sn-glycero-3-phosphorylcholine (SONPC) *m/z* 678), and the isomers of PAPC, PLPC, SAPC, and SLPC hydroxides and hydroperoxides (PAPC-OH, PAPC-OOH, PLPC-OH, PLPC-OOH, SAPC-OH, SAPC-OOH, SLPC-OH, and SLPC-OOH) were identified as in (Gruber et al. 2012). Extraction comparability between the groups was controlled by calculating the relative intensity of the spike-in standard di-nonanyl-ch-glycero-3-phosphorylcholine (DNPC) to DPPC (Supplementary Fig. 1). Dot plots of relative intensities were generated with GraphPad Prism 8 software. The statistical significance of differences between the groups was determined with un-paired two-tailed Student’s *t* test and significant differences are indicated by asterisks (*p < 0.05; **p < 0.01; ***p < 0.001; NS—no significant differences). Pearson correlation was used for correlation analysis between relative lipid intensities and the RMIs.

### Autofluorescence images and image analysis

OCT compound embedded rat frontal cortices were cut to 6 µm-thick cryo-sections, fixed with acetone at – 20 ℃ for 10 min, and washed with phosphate-buffered saline, and nuclei were stained with Hoechst reagent. Images were acquired with an Olympus BX63 fluorescence microscope equipped with a UC90 camera and operated through the cellSens software (all Olympus, Hamburg, Germany) at ×4 and ×40 magnification. Images were acquired in the DAPI, FITC, brightfield (BF) and differential interference contrast (DIC) channels. Where indicated z-stack images were acquired at a step size of 1 µm and subsequently merged to an extended focus imaging. Light source and camera settings for individual channels and magnifications were maintained unchanged throughout the experiment. To investigate differences in autofluorescence intensities of the different samples in the FITC channel 40 × images from three different rats per group were analyzed with the StrataQuest context analysis software (version 7.0.1.165, TissueGnostics GmbH, Vienna, Austria) (Ecker and Steiner 2004). Nuclei were detected by the StrataQuest “nuclear segmentation” engine (version2) and measurement masks were grown around the detected nuclei with a maximum radius of 5 µm utilizing the StratQuest “cellular mask” engine (version 2). Single-cell measurements of FITC intensity [8-bit] were determined for each animal and each group. Boxplots were generated displaying color-coded high-intensity outlier events (values above 75th percentile + 1.5 × interquartile range (IQR)) and statistical analysis was performed in RStudio version 2021.09.1 + 372 “Ghost Orchid” Release (RStudio: Integrated Development Environment for R. RStudio, PBC, Boston, MA). The statistical significance of differences between the groups was determined with un-paired, two-tailed Wilcoxon rank sum test and significant differences are indicated by asterisks (***p < 0.001; NS—no significant differences).

## Results

**Fig. 1:**
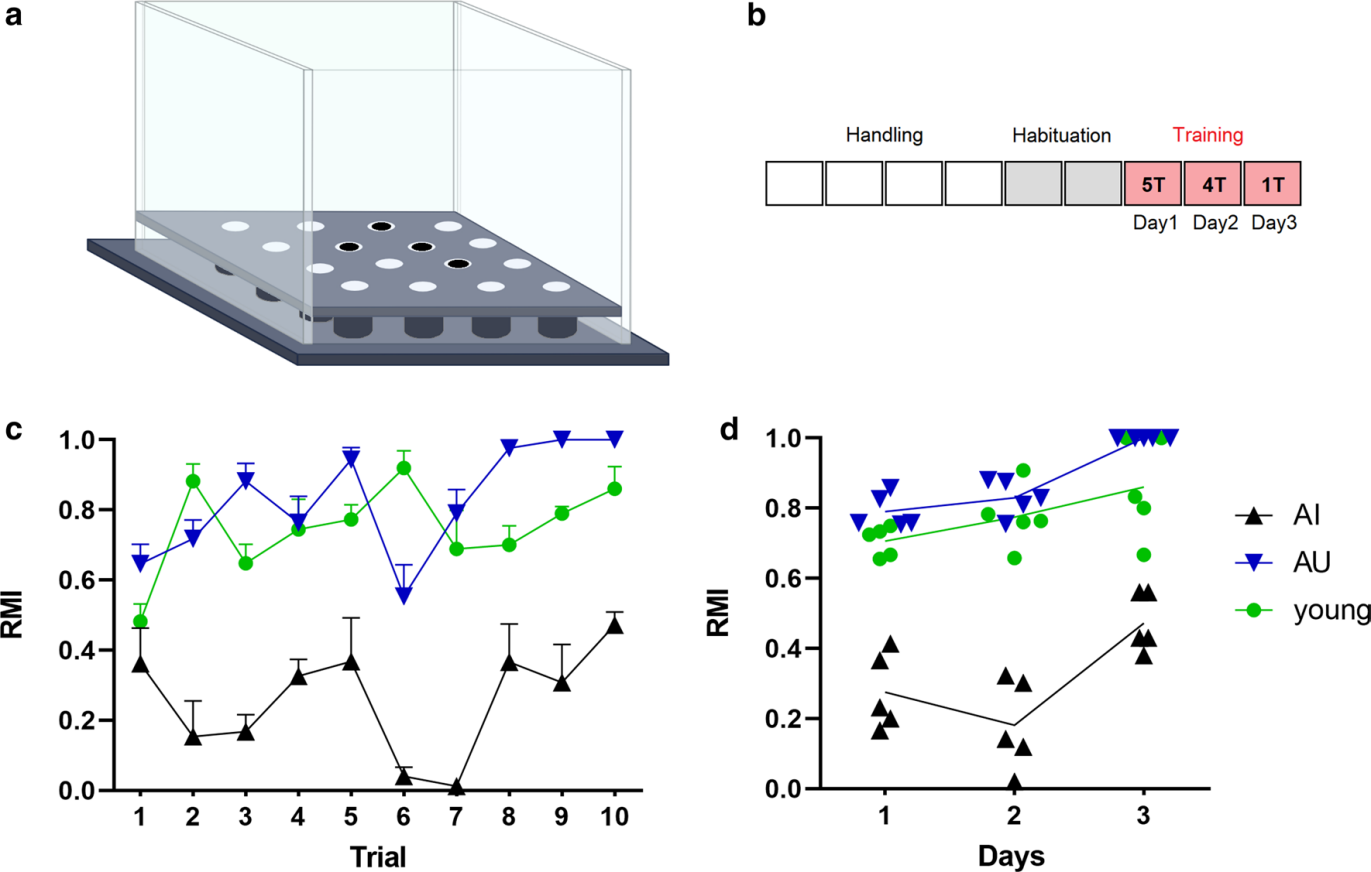
Performance of young and aged rats in spatial learning and memory task. Schematic demonstration of hole-board (**a**) and experimental protocol (**b**). **c**, **d** Performance of young (green circles), and old, cognitively unimpaired (AU, blue triangles) and impaired (AI, black triangles) male rats. Both, young and AU exhibited higher RMI compared to AI rats over 3 days of training in the hole-board test. Statistics are detailed in the results section. Values are expressed as mean ± SEM, *n* = 5 for each group

Learning and memory performance of aged rats was evaluated in the hole-board test (Fig. 1a, b). Five rats characterized as either impaired (AI) or unimpaired (AU) performers based on their mean reference memory index (RMI) derived from performance on day 2 (trial 6–9) and day3 (trial10) were randomly selected from a large cohort of aged rats (*n* = 127). The behavioral results are shown in Fig. 1c and d. AI rats performed significantly worse than AU (two-way RM-ANOVA, day 1 [*F*(1,8) = 94.43, *P* < 0.0001), day 2 (*F*(1,8) = 110, *P* < 0.0001); un-paired Welch`s test, day 3 (*t* = 14.24, *P* = 0.0001)] and young rats [two-way RM-ANOVA, day 1 (*F*(1,8) = 68.51, *P* < 0.0001), day 2 (*F*(1,8) = 72.03, *P* < 0.0001); un-paired *t* test, day 3 (*t* = 5.271, *P* = 0.0008)]. AU animals performed slightly better than young animals [two-way RM-ANOVA, day 1 (*F*(1,8) = 8.758, *P* < 0.0182], day 2 [*F*(1,8) = 1,445, *P* = 0.2637); un-paired Welch`s test, day 3 (*t* = 2.201, *P* = 0.0925)].

Oxidized phospholipids (OxPLs) can be divided into two structural groups according to the integrity of the fatty acid moiety carbon chain in comparison to the non-oxidized precursors. First, the non-fragmented OxPLs with the same chain length of the fatty acid as in the precursor phospholipid, which include phospholipid hydroperoxides, -hydroxides, and second, the oxidatively fragmented OxPLs that have shortened FA chains. The latter group includes the azelaoyl- and oxo-nonanoyl phospholipids and the lysophospholipids.

**Fig. 2:**
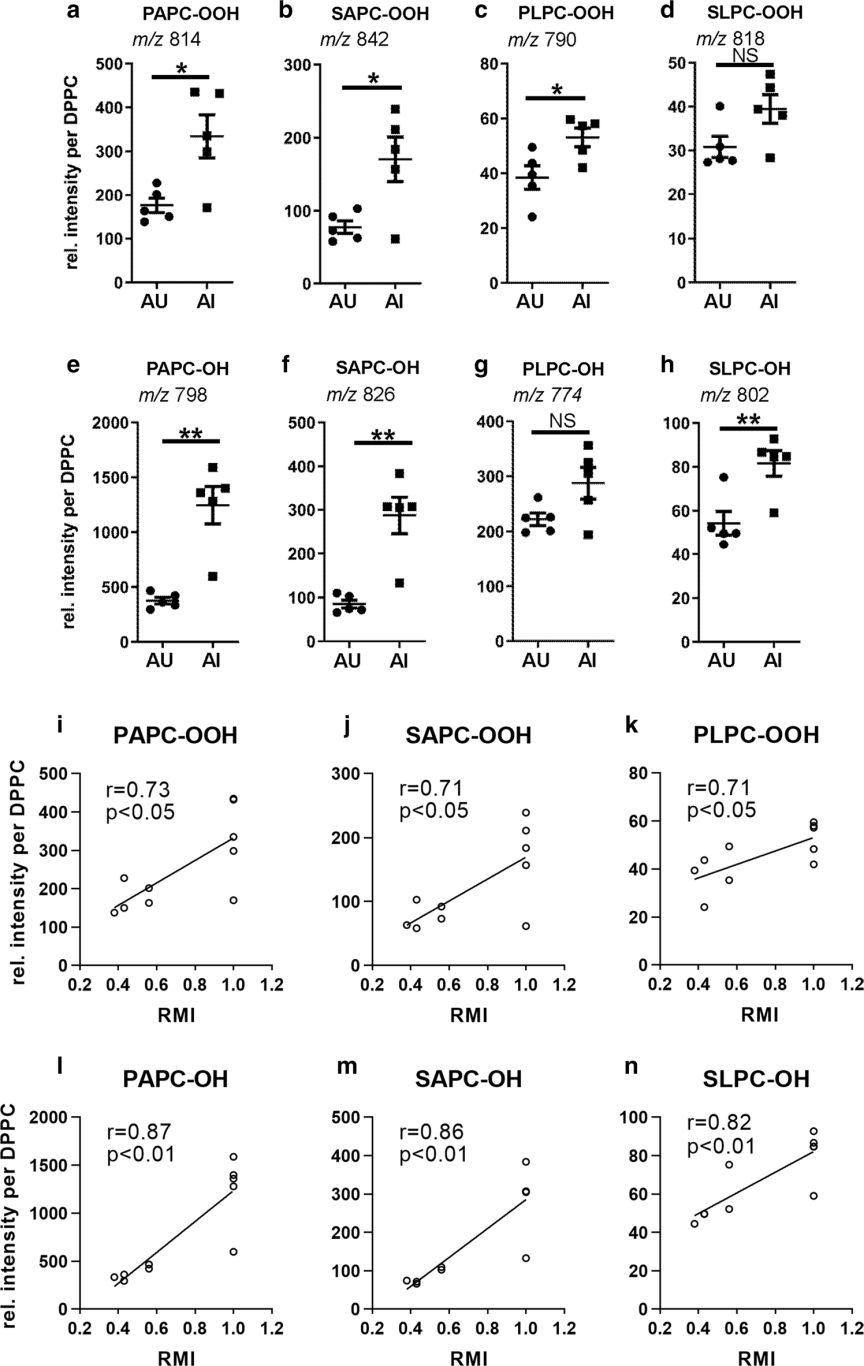
Comparison of oxidized phosphatidylcholine (PC) in the frontal cortex of memory unimpaired and impaired aged rats. Polar lipids were isolated from the frontal cortex of aged rats with unimpaired (AU) and impaired memory (AI). PC hydroperoxide species were quantified with HPLC MS/MS. Dot plots show the abundance of the respective PC-OOH (**a–d**) and PC-OH (**e–h**) species extracted from AU and AI rat frontal cortex normalized to DPPC as internal standard (*n* = 5; error bars indicate SEM) and relative abundance values were multiplied by 10.000 to present data in numbers larger than one. Asterisks indicate statistically significant differences (*p < 0.05; **p < 0.01; *NS* no significant differences) determined by Student’s *t* test. **i–n** Pearson correlation analysis revealed a positive correlation between relative lipid intensities and memory performance on the last day of the hole-board test

When we compared the levels of phosphatidylcholine hydroperoxides (PC-OOH) in the frontal cortex of memory impaired aged rats to those in unimpaired aged rats using an HPLC–MS/MS OxPL-screening method (Gruber et al. 2012) and normalizing to a non-oxidizable internal control (DPPC), we found moderate elevation of PC hydroperoxides (Fig. 2a–d). Those PC-OOH that derived from arachidonic acid oxidation showed higher relative increase in abundance than the linoleic acid-derived PC-OOH. The PC-OH were elevated higher than the PC-OOH species, especially those derived from arachidonic acid, which correspond to PC-esterified HETE. (Fig. 2e–h). Furthermore, across individual animals, we found significant positive correlations between the performance of AU and AI rats on the last day of hole-board test and relative lipid intensities (Fig. 2i–n).

**Fig. 3:**
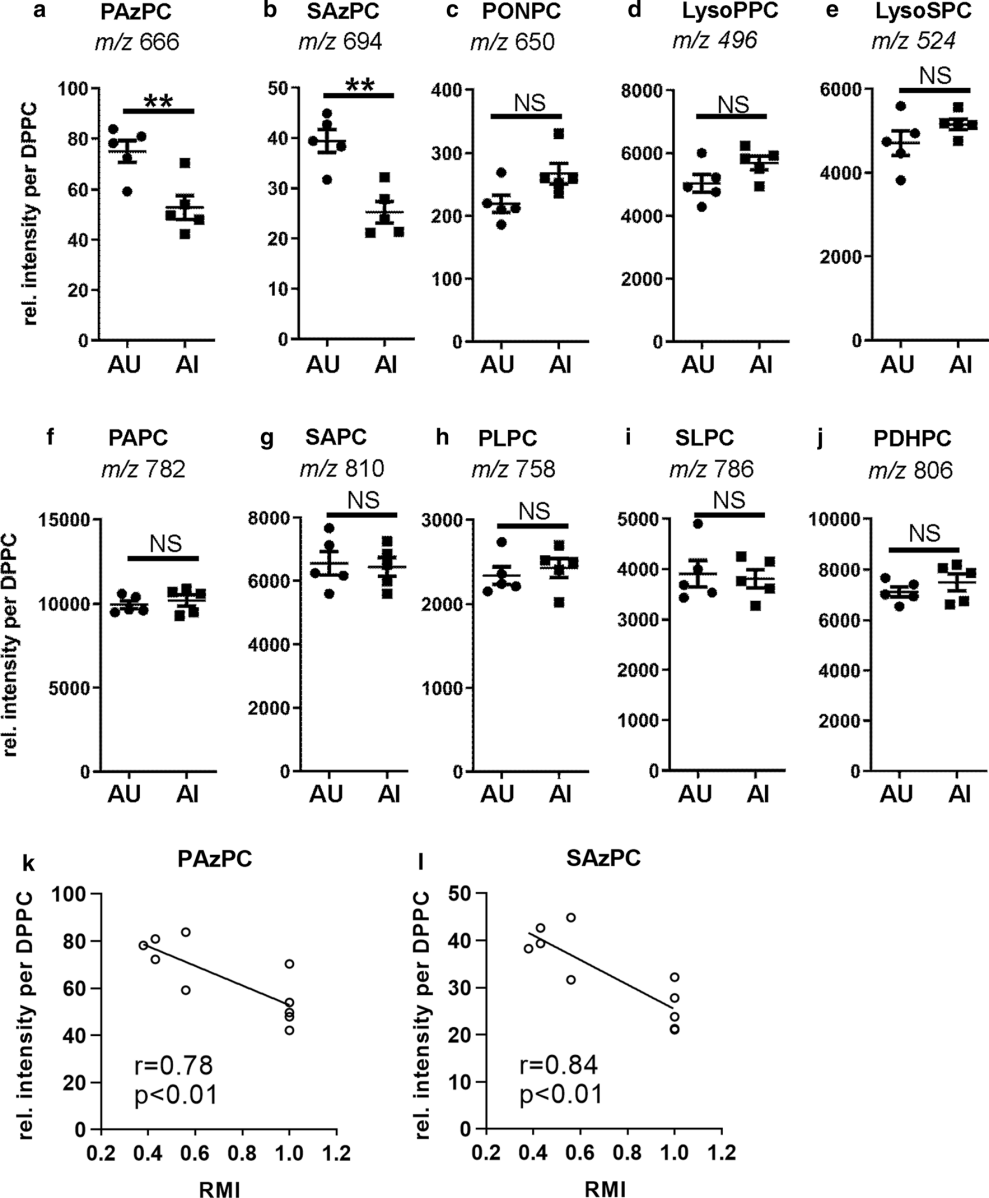
Comparison of native, non-oxidized and lipid species resulting from oxidative fragmentation of oxidized fatty acid moieties in the frontal cortex of memory unimpaired and impaired aged rats. Polar lipids were isolated from the frontal cortex of aged rats with unimpaired (AU) and impaired memory (AI). Fragmented lipid and non-oxidized species were quantified with HPLC MS/MS. Dot plots show the abundance of the respective azelaoyl (**a**, **b**), terminally carbonylated (**c**), lysophospholipid (**d**, **e**), and native, non-oxidized phospholipid (**f–j**) species extracted from AU and AI rat frontal cortex normalized to DPPC as internal standard (*n* = 5; error bars indicate SEM). Asterisks indicate statistically significant differences (**p < 0.01; *NS* no significant differences) determined by Student’s *t* test. **k–l** Pearson correlation analysis revealed a negative correlation between relative lipid intensities and memory performance on the last day of the hole-board test

The chain shortened OxPL species are advanced oxidation products that result from oxidative fragmentation of the oxidized fatty acid moieties by various mechanisms, including β-scission and Hock cleavage (Bochkov et al. 2010; Gugiu et al. 2006). When we analyzed fragmented lipid species, we found, surprisingly, that the azelaoyl-PC species (esterified di-carboxylic acids) were significantly decreased in the aged memory impaired group (Fig. 3a, b). As shown in Fig. 3 k–l, relative lipid intensities of PAzPC and SAzPC negatively correlated with RMIs on the last day of training. This is of interest as we had found PAzPC and SAzPC elevated in brains of autophagy-deficient mice, which had displayed signs of neurodegeneration at 1 year of age, as compared to their autophagy competent siblings. Another chain shortened lipid species, the terminal carbonyl containing PONPC, was, however, not significantly changed when comparing memory impaired and unimpaired groups (Fig. 3c). Next, we investigated the lysophospholipid species. These lipids are both the endproducts of non-enzymatic phospholipid peroxidation but also products of phospholipases that cleave the fatty acid chain. They serve also as important substrates for re-esterification of fatty acids in phospholipid synthesis. Lyso-palmitoyl-PC (Lyso-PPC) and Lyso-stearoyl-PC (LysoSPC) levels were not significantly changed between the impaired and unimpaired memory groups (Fig. 3d, e).

Also not changed were the levels of the native, non-oxidized phospholipid species (Fig. 3f–j). PAPC and SAPC give rise to PAPC or SAPC hydroperoxides and -hydroxides, respectively. PLPC and SLPC give rise to the respective hydroperoxides and -hydroxides as well as to the azelaic acid and oxononanoic chain shortened oxidation products.

**Fig. 4:**
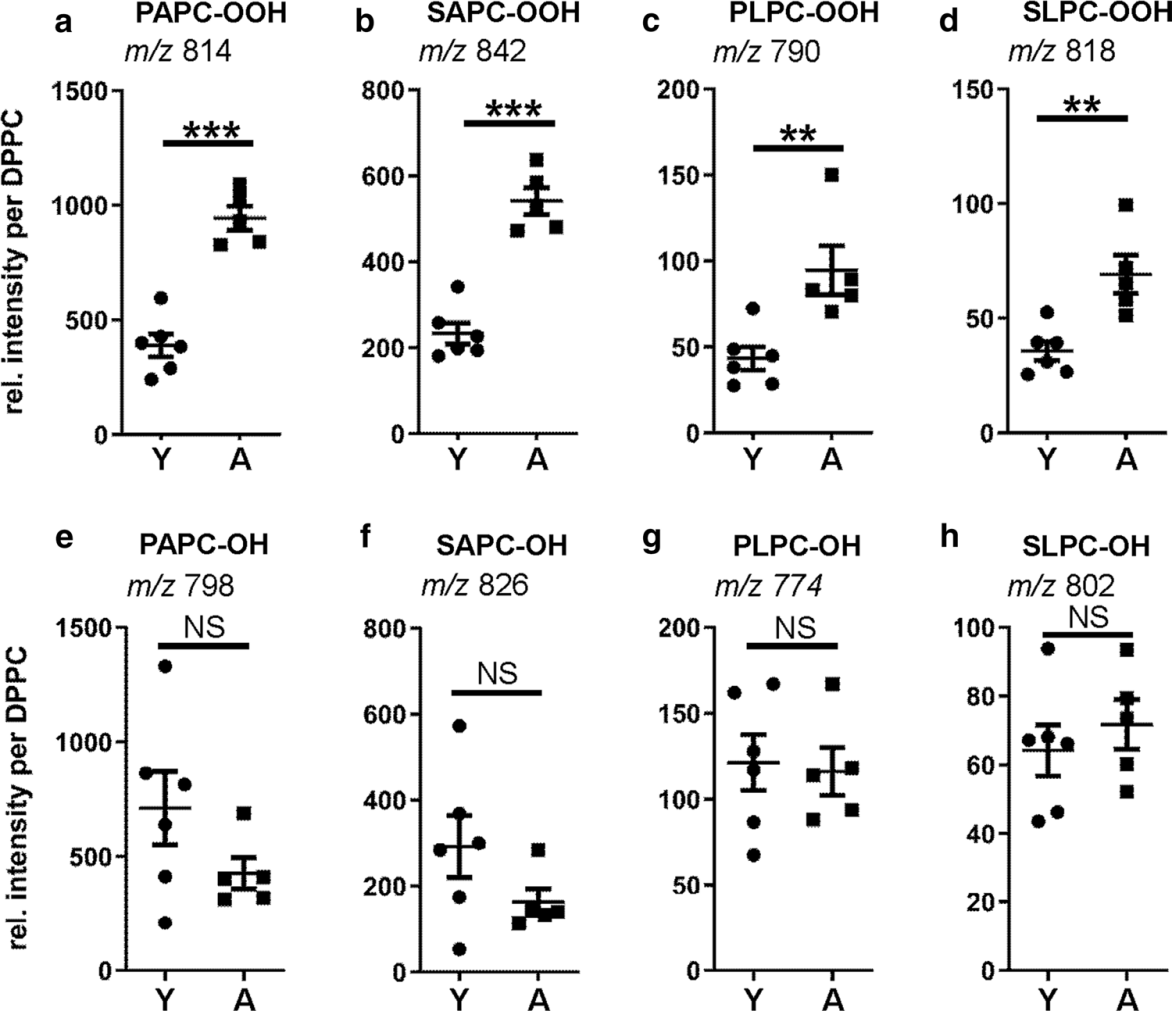
Impact of chronological age on the composition of phospholipids in the rat frontal cortex. Polar lipids were isolated from the frontal cortex of young (Y) and aged (A) rats. Non-fragmented oxidized PC species were quantified with HPLC MS/MS. Dot plots show the abundance of the respective PC-OOH (**a-d**) and PC-OH (**e–h**) species extracted from the frontal cortex of young and aged rats normalized to DPPC as internal standard (young *n* = 6, old *n* = 5; error bars indicate SEM). Asterisks indicate statistically significant differences (**p < 0.01; ***p < 0.001; *NS* no significant differences) determined by Student’s *t* test

Next, we investigated how chronological age would impact the composition of phospholipids and their oxidation products in rat frontal cortex. We found a significant age-dependent relative increase in all investigated classes of phospholipid hydroperoxides, which was more pronounced than the difference between impaired and unimpaired aged animals (Fig. 4a–d). The phospholipid hydroxide levels, however, were not significantly different between the age groups (Fig. 4e–h).

**Fig. 5:**
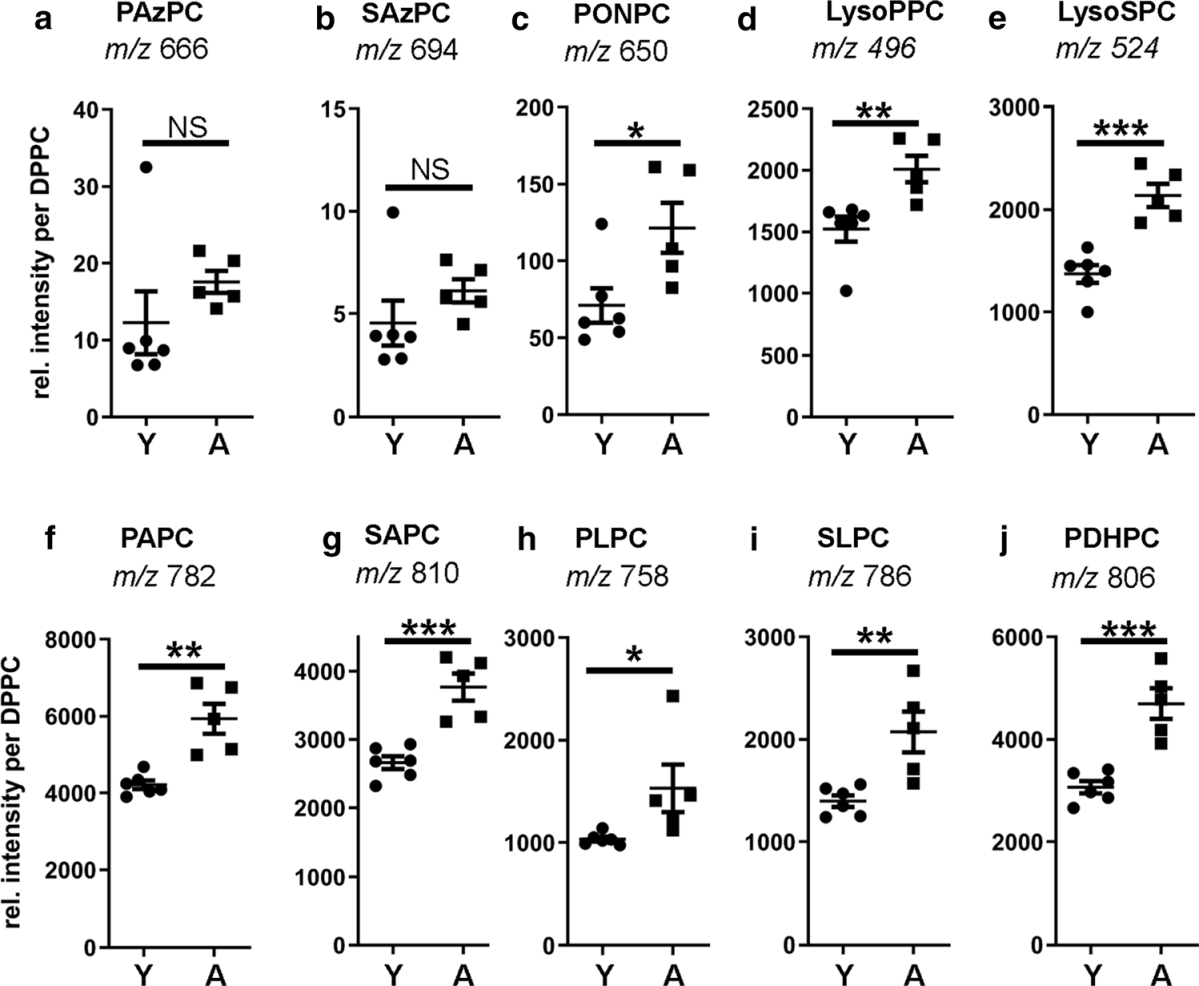
Impact of chronological age on the composition of native, non-oxidized and lipid species resulting from oxidative fragmentation of oxidized fatty acid moieties in the frontal cortex young and aged rats. Polar lipids were isolated from the frontal cortex of young and aged rats. Fragmented and non-oxidized lipid species were quantified with HPLC MS/MS. Dot plots show the abundance of the respective azelaoyl (**a**, **b**), terminally carbonylated (**c**), lysophospholipid (**d**, **e**), and native, non-oxidized phospholipid (**f-j**) species extracted from the frontal cortex of young (Y) and aged (A) rats normalized to DPPC as internal standard (young *n* = 6, old *n* = 5; error bars indicate SEM). Asterisks indicate statistically significant differences (*p < 0.05; **p < 0.01; ***p < 0.001; *NS* no significant differences) determined by Student’s *t* test

When analyzing the fragmented lipid species of young and aged rat brains, we found that the azelaoyl PCs were not significantly different (Fig. 5a, b), whereas PONPC, an aldehydophospholipid, was significantly elevated in aged brains (Fig. 5c). Also elevated were the investigated lysophospholipid species (Fig. 5d, e). Furthermore, we found a general elevation of the native, un-oxidized phospholipid classes with polyunsaturated fatty acid moieties, with the polyunsaturated, arachidonic or docosahexaenoic species showing a higher age-dependent increase (Fig. 5f–j).

**Fig. 6:**
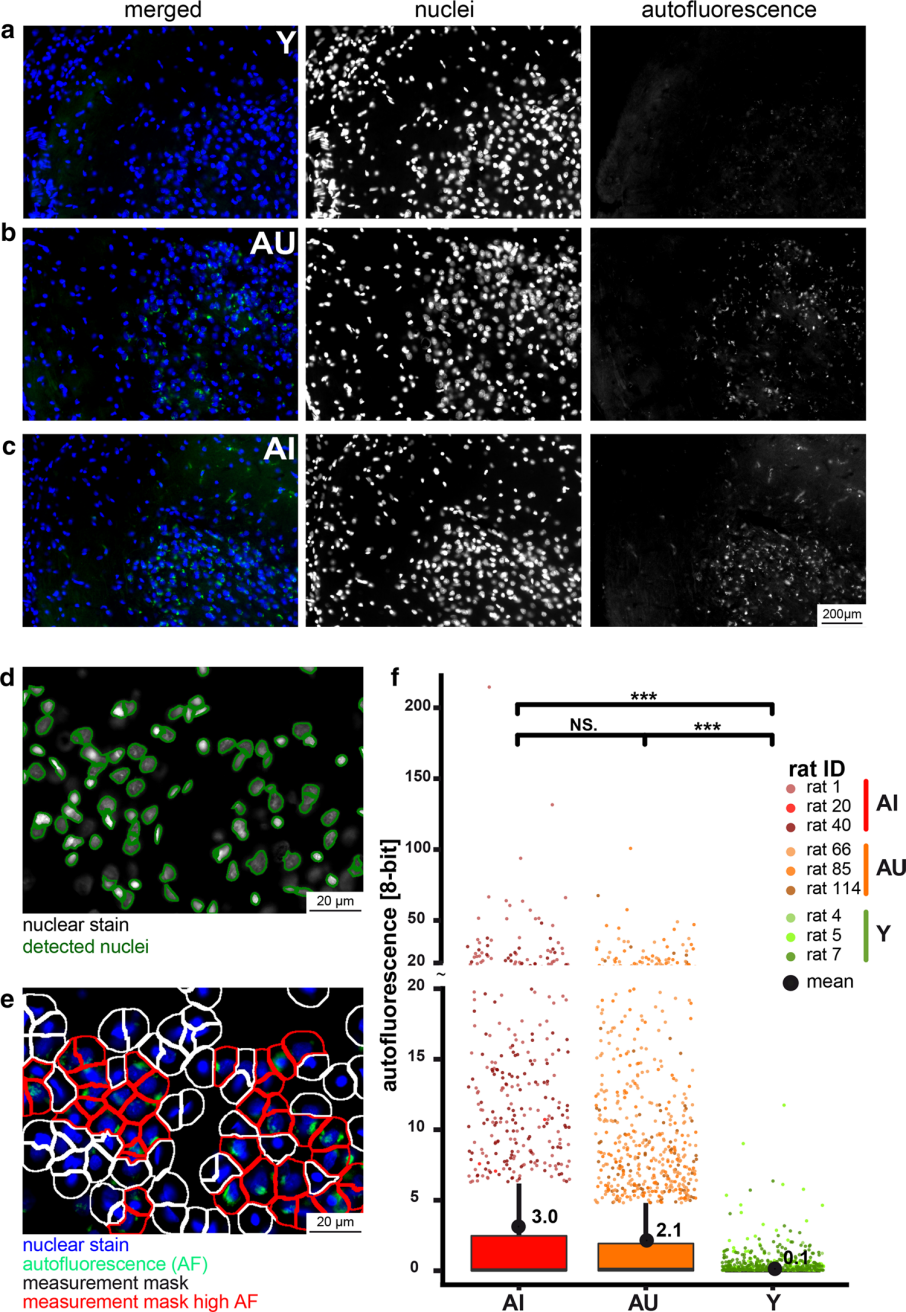
Comparison of autofluorescence in frontal cortex sections of young, aged unimpaired and impaired rats. Cryo-sections of the frontal cortex of young (Y), aged unimpaired (AU) and aged impaired (AI) rats were counterstained with Hoechst reagent nuclear dye and images of the nuclei and the autofluorescence were acquired. Upper panel: merged, nuclear- and autofluorescence images of Y (**a**), AU (**b**), and AI (**c**) frontal cortex. Lower panel: quantification of bright autofluorescence aggregates. Automatically detected nuclei are indicated by green boundaries (**d**), predicted cell masks indicating measurement areas are indicated with white or red boundaries indicating signal below or above the 75th percentile + 1.5 × IQR, respectively (**e**). Boxplots depicting single-cell measurements of autofluorescence intensity within the measurement masks, hinges correspond to the 25th and 75th percentile, upper whisker extends to 75th percentile + 1.5 × IQR. Dots represent cells containing bright autofluorescent aggregate spots within each group and are color-coded for individual rats (*n* = 3 per group). Mean values are indicated by black dots. Asterisks indicate statistically significant differences (***p < 0.001; *NS* no significant differences) determined by Wilcoxon rank sum test

Aldehydophospholipids are highly reactive and can modify and crosslink proteins. Lipids are a major component of lipofuscin, an autofluorescent lipopigment frequently correlated with central nervous system (CNS) aging and age-related neurodegeneration (Moreno-Garcia et al. 2018). Lipofuscinogenesis has been found the investigation of human frontotemporal degeneration (Smith et al. 2012). Thus, we investigated cryo-sections of prefrontal lobe of the young, the old memory unimpaired and impaired individuals for autofluorescence with advanced imaging processing software. Indeed, in the old rats, irrespective of their hole-board performance, we could observe significantly elevated autofluorescence in the perinuclear pattern characteristic for brain lipofuscin deposits (Moreno-Garcia et al. 2018) (Fig. 6a–f and supplementary Fig. 2).

## Discussion

A major finding of this study was that decreased performance of aged rats in spatial learning tests was reflected in the composition of oxidized phospholipids isolated from their (prefrontal) cortex tissue. The data show that the observed oxidation pattern was not a mere exacerbation of age-related changes or a general increase in phospholipid oxidation. Chronological aging led to elevation of phospholipid hydroperoxides, of lysophospholipids and of the aldehydophospholipid PONPC. Also, the un-oxidized (poly-)unsaturated phospholipids were elevated in comparison to di-palmitoyl-PC (DPPC), a phospholipid with saturated FA and thus not oxidizable under these conditions. Impaired memory performance in old animals was paralleled by not only a further increase in PC hydroperoxides, but also with strong elevation of the hydroxy-modifications of PC, especially those deriving from arachidonic acid. Furthermore, azelaoyl-PC species were significantly decreased in the memory impaired rats. Both, PC hydroxides and azelaoyl-PC levels had not been affected by chronological aging. These lipid oxidation patterns can only partially be explained by ROS-mediated lipid peroxidation that would be low in young, elevated in unimpaired and even more elevated in aged, memory impaired brains. A mere gradient of lipid peroxidation in these groups could explain the relative increases in PC-OOH. Non-enzymatic further oxidation would, however, lead to generation of PC-OH, as we have shown for singlet oxygen-mediated peroxidation of PAPC in vitro (Gruber et al. 2007). Thus, there must be a mechanism that limits the relative abundance of PC-OH in the unaffected aged brains, while the aldehydophospholipid PONPC and the lysophospholipids are elevated.

The memory impaired rats, on the other hand, showed a strong elevation of the PC-OH; thus, either the mechanism limiting the PC-OH was inactive, or another mechanism that elevated PC-OH, and especially the hydroxides of arachidonate-PC, was activated. Again, simple peroxidation does not explain why the azelaoyl-PC species, which would result from (auto-)oxidation of linoleoyl-PCs, would be decreased. While bona-fide identification of the mechanisms behind these unexpected and novel findings will need further studies, we here discuss which known mechanisms could be involved in generating this OxPC pattern and we discuss how our HPLC–MS/MS data compare to earlier findings by others on phospholipid composition and its age- or pathology-related changes in mammalian brain.

### PUFA metabolism in aging brain

The turnover of AA and DHA in rat and human brain is very high, with around 5% per day; thus, it is believed that in healthy brain, the PUFA content, which is important for brain function, can be rapidly and permanently balanced (Rapoport et al. 2001). The human prefrontal cortex is the brain region with the highest content of PUFAs and the exploration of the exact molecular species present in specific regions of the brain with modern high-sensitivity and high-resolution MS lipidomics and MSI is in its early stages [Rev. in (Pamplona et al. 2019)]. Earlier studies have found a decrease in (esterified or free) AA and DHA, in late stages of the aging of human and rat brains. One early study found age-related decrease in PUFA phospholipid content [especially 22:6 (n-3) and 22:4 (n-6) for the phosphatidyl-ethanolamine (PE), phosphatidylinositol (PI) and phosphatidylserine (PS)] but not PC in the cerebral cortex (Lopez et al. 1995). Other studies again have found increased AA content of phospholipids in aging rat tissue (Tamburini et al. 2004). The vast majority of studies so far did not investigate the specific molecular species in defined regions of rat brains, and thus, our findings on increased levels of PUFA-PC to a saturated PC species using a semi-targeted HPLC–MS/MS lipidomic method (Gruber et al. 2012) may elucidate subtle differences in the age-dependent dynamics of PUFA esterified to different PL backbones that so far went unnoticed.

A role for PL-esterified AA in nervous signal transduction involving the glutamate receptor and protein kinase C (PKC) activation has been reported (Nishizuka 1984) and will be target of further investigation.

### PL oxidation in aging and age-related tissue decline

Aging of tissues, including the brain, is frequently associated with elevated redox stress, which can be caused by accumulation of dysfunctional mitochondria, impaired redox responses, impaired repair capacity, changes in metabolism, and chronic inflammation. Especially, proteins of the energy metabolism, the cytoskeleton, and such involved in neurotransmission appear to be susceptible to lipoxidation with the reactive lipid species accumulating in age (Pamplona et al. 2019). The human prefrontal cortex (PFC) is also the region of the brain with the highest peroxidizability index, a measure for theoretical susceptibility to lipid peroxidation (Naudi et al. 2017).

### Phospholipid hydroperoxides and phospholipid hydroxides

Oxidation of the unsaturated FA moieties of PL can proceed as a non-enzymatic process that is mediated nonspecifically by ROS which originate from mitochondria and are increased in aging. Alternatively, oxidation can be mediated by specific enzymes which are dysregluated in aging and "inflammaging", the low-grade inflammatory phenotype often going in hand with aging. These enzymes include lipoxygenases and cyclooxygenases, and cytochrome p450 which all act primarily on free PUFA, and usually require re-esterification of the oxidized PUFA to the PL backbone by enzymes including the fatty acyl CoA ligase (FACL). However, the primate 12/15-lipoxygenase and the rodent 15-lipoxygenase can generate PL-esterified hydroperoxides which are reduced to hydroxides by phospholipid hydroperoxide glutathione peroxidases (PHGPx) (O'Donnell et al*.* 2012). The modulation of enzymes including peroxiredoxin 6 (PRDX6) and of phospholipases will also influence the steady-state levels of PL-esterified hydroxy fatty acids other than eicosanoids in cells. PRDX6 has a GPx activity that can reduce hydrogen peroxide and short-chain fatty acid hydroperoxides (FA–OOH) and also phospholipase, and lysophospholipid acyltransferase activities (Fisher et al. 2016). We have recently shown that in skin of mice, transgenic overexpression of peroxiredoxin 6 reduces the levels of PC-OOH, while gene knockout leads to their elevation in vivo (Rolfs et al. 2013). Importantly, we have recently found that in a comparable cohort of rats, PRDX6 was decreased in the dentate gyrus brain region of rats with impaired memory (Lubec et al. 2019). Reduced expression or activity of peroxiredoxin 6 may thus also explain the elevation of PL-OOH in the PFC; however, neither the peroxidase nor the phospholipase function of this enzyme appears to influence the OxPC levels, as one could in that case expect a decrease of PC-OH and lysophospholipids, respectively. In Alzheimer’s disease patients, the erythrocyte PC-OOH levels were found elevated (Yamashita et al. 2016) indicating an association of phosphatidylcholine peroxidation with neurodegenerative disease. Both PC-OH and PC-OOH can induce cell adhesion to ICAM-1 (Arai et al. 1997), and activate the NRF2-dependent antioxidant response (Jyrkkanen et al. 2008) and the unfolded protein response (Gargalovic et al. 2006). If PL-OOH are not detoxified at some point, they can induce cell death following the peroxidation chain reaction (Imai et al. 2017), and this so-called ferroptosis is a candidate mechanism that could promote neurodegeneration and neuro-inflammation (Maiorino et al. 2018).

The enzymes able to modulate the levels of oxidized PUFA attached to PC which have reported effects on brain function are the lipoxygenases. Inhibition of or deficiency in Alox15, which is required for production of resolvin D1 from DHA (22:6; n-3) and is strongly expressed in the prefrontal cortex, reduces performance in the T maze test (Shalini et al. 2018). On the other hand, inhibition of 5-lipoxygenase protects dopaminergic neurons (Kang et al. 2013), and 12 and 15 HETE are elevated in a mouse model of Amyotrophic Lateral Sclerosis (ALS) (Trostchansky et al. 2018). The Alox15 gene product (12/15-lipoxygenase) can metabolize PUFA esterified to phospholipids, and interestingly, the resulting phospholipid oxidation products appear to have an important immunomodulatory and anti-inflammatory function. Genetically engineered lack of this enzyme in mice aggravates central nervous system inflammation in the experimental autoimmune encephalomyelitis (EAE) model, and this phenotype goes in hand with impaired silencing of pro-inflammatory signaling in dendritic cells through oxidized phospholipids in these mice, as we have shown (Rothe et al. 2015). Thus, for both free and esterified phospholipid hydroperoxides, -hydroxides, and the enzymes that regulate their abundance, no generalizable effect can be attributed, but very specific studies are needed to find causal and local relationships in aging and neurodegeneration. Therefore, the observed increase in PC-OOH and strong increase of PC-OH in aged memory impaired as compared to aged unimpaired is likely the result of a complex interplay of non-enzymatic and several enzymatic pathways. The generated bioactive lipids, however, have the capacity to induce and promote major effects in neuroinflammation, protein folding, and stress signaling that could contribute to the impaired memory phenotype.

### Aldehydophospholipids

Reactive carbonyl species that derive from non-enzymatic peroxidation of PUFA and PUFA moieties can form advanced lipoxidation products (ALE) with other macromolecules (Domingues et al. 2013; Naudi et al. 2017). A characteristic type of ALE is the autofluorescent granular pigment termed lipofuscin, and the composition of brain lipofuscin is similar in human and rat (Ottis et al. 2012). This poorly soluble material, composed of mostly proteins and adducted lipids, is found in neurons and frequently associated with aging. In both groups of aged rats, irrespective of their learning and memory performance, we detected massive autofluorescence indicative of lipofuscin, and interestingly, both groups showed significantly elevated PONPC, a typical reactive carbonyl lipid species capable of covalently modifying proteins (Bochkov et al. 2010). The accumulation of lipofuscin is not only a marker but a potential promotor of neurodegeneration (Moreno-Garcia et al. 2018), and the age-related formation of aldehydophospholipids in the PFC might contribute to its formation.

### Azelaoyl PCs

PAzPC and SAzPC, two fragmented oxidized lipid species that were by trend elevated (albeit not significantly) in the aged cohort were strikingly and significantly less abundant in the memory impaired individuals. We have previously described the PL-esterified azelaic acid as redox stress inducible in epidermal keratinocytes (Narzt et al. 2019) and dermal fibroblasts (Gruber et al. 2012), and as significantly elevated in autophagy-deficient, senescent melanocytes (Ni et al. 2016) and in autophagy-deficient keratinocytes (Zhao et al. 2013). Interestingly, we recently found these species significantly elevated in the brains of mice in which autophagy was deleted in tyrosinase expressing cells. The knockout mice showed signs of increased neurodegeneration upon aging (Sukseree et al. 2018). Interestingly, in several human brain disorders, elevated urinary azelaic acid serves as disease biomarker, and it remains to be elucidated whether there could be a direct causal relationship with decreased esterified azelaic acid in the brain phospholipids and an increase in urinary free azelaic acid in neurological disease or neurodegeneration (An and Gao 2015). The surprising finding that the AzPC species are decreased may thus be explained by dysregulated autophagy, which itself is emerging as essential for memory formation (Glatigny et al. 2019). Antibodies detecting azelaoyl-PC but also PONPC have been found in the cerebrospinal fluid of multiple sclerosis patients (Kanter et al. 2006).

Our observation on the decreased levels of PL-esterified azelaic acid in the memory impaired animals, while most other lipid species were elevated cannot be explained without further investigation, but it allows one conclusion. That the levels of oxidized phospholipids in the impaired vs the unimpaired brains do not simply reflect the result of ROS-mediated PL peroxidation, because in such a scenario, also PAzPC and SAzPC would be elevated as we demonstrated earlier. The decreased levels of esterified AzA could hypothetically be due to increased modification (e.g., oxidation, as AzA has been reported an ROS scavenger), metabolization (e.g., specific cleavage by phospholipases), or degradation (e.g., via autophagy) of the AzPCs in the context of the impaired brains, but all of those possibilities would have to be tested experimentally. How the decreased free or esterified AzA could contribute the worsened aging phenotype may be connected to reduced anti-inflammatory PPARγ activation (PMID: 23278893) or favorably regulating fatty acid metabolism in aging (PMID 33449249), but the limited existing studies on AzA have not been made on the brain and its aging.

### Lysophospholipids

Lysophospholipids are the final oxidation product of phospholipid autoxidation or cleavage of phospholipids by phospholipases, and serve as a substrate for synthesis of new phospholipids by acyltransferases. Lyso-PC have been found elevated in aged tissue, and the most likely reason for their elevation is an age-related increase in PLA2 activity (Kim et al. 1997). Another study found a correlation of lysophospholipids of the rat PFC with the blood levels of corticosterone in a stress model (Oliveira et al. 2016).

## Conclusion

**Fig. 7:**
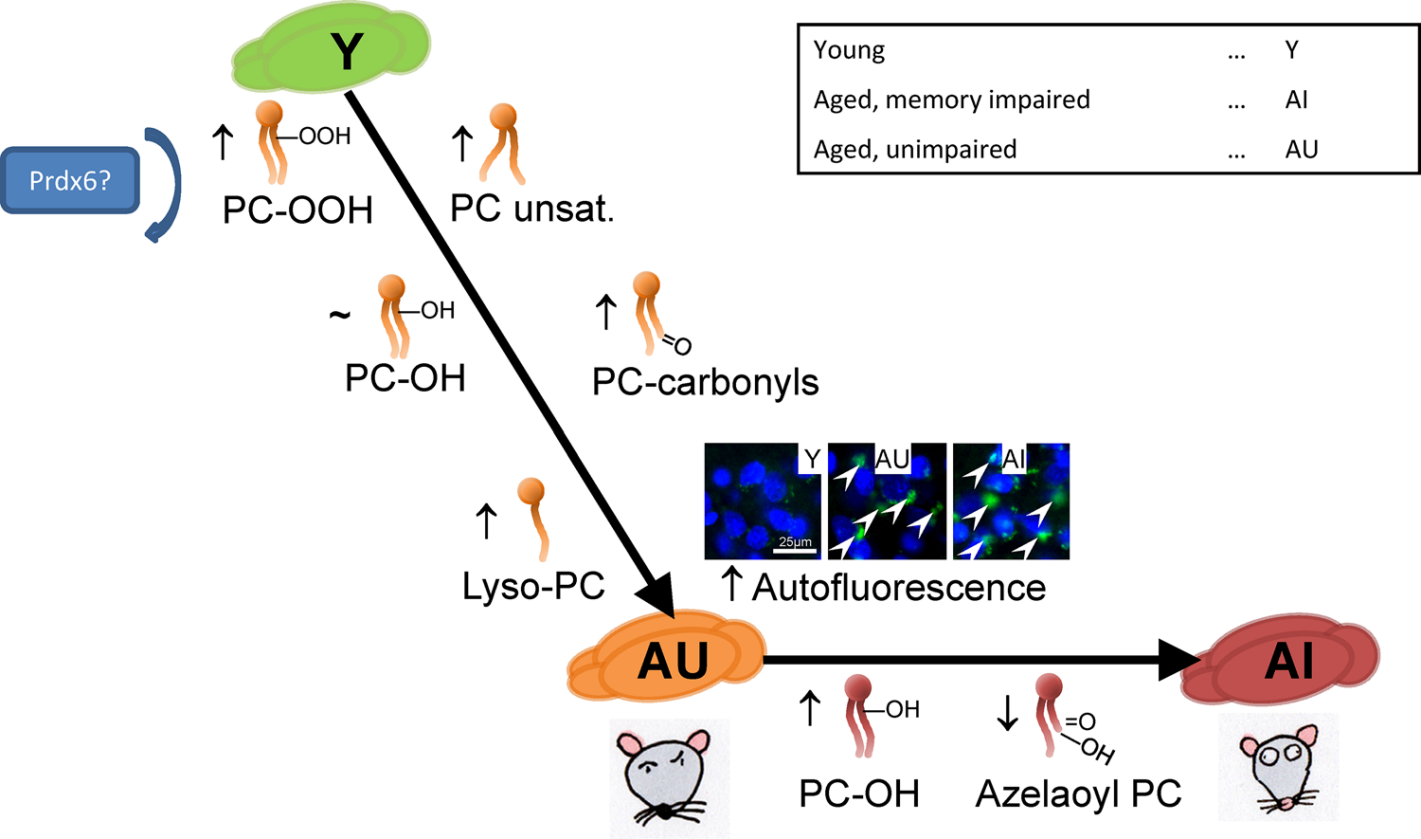
Schematic depiction of changes in the redox lipidome of aged rats. Age-related changes included increase in unsaturated PC, PC-OOH, Lyso-PC, PC-carbonyls, and in cortical autofluorescence. In aged, memory impaired brains, there was an increase in PC-OH and a decrease in azelaoyl-PC

Together, these results identify specific oxidized phospholipid species that are elevated in the PFC of aged rats, and a distinct group of lipid species elevated in PFC of rats with impaired learning performance. These findings are compatible with a concept of age-related, mostly ROS driven increase in ongoing lipid peroxidation which manifests in elevated levels of phospholipid hydroperoxides. The further elevation of phospholipid hydroxides that was exclusively observed in the aged, memory impaired group could be an indication of an additional (neuro-) inflammatory component in the learning-impaired cohort (Fig. 7). The cause of the observed heterogeneity regarding spatial memory scores within the groups is a finding that merits further studies. One topic of intense investigation which could be connected to the functional heterogeneity we observed is the heterogeneity of senescent cells and their senescence-associated secretory phenotype (SASP) (Zit Gasek et al. PMID: 34841261). This heterogeneity, which is believed to result from either, diverse initial damaging events that induced senescence, or from epigenetic events influencing the senescence process (Zit Nacarelli PMID: 29186801), would then be amplified by the corresponding (and equally heterogeneous) SASP. In that hypothesis distinct, stochastic events causing cellular senescence during the lifespan could cause and amplify heterogeneous downstream functional effects of aging even within a genetically uniform cohort. As we have recently identified some of the OxPC investigated here as being associated with cellular senescence in the skin and as potential SASP factors (PMID: 33333126), further studies would have to identify whether it is senescent cells that are source of lipids regulated in aging and whether their number and distribution would be correlated with memory impairment. Our findings suggest that targeting the brain lipidome with dietary or pharmacological interventions may affect the learning performance in mammals.

## Supplementary Information

Below is the link to the electronic supplementary material.

Supplementary file1 (TIFF 283 KB)

Supplementary file2 (TIF 7682 KB)
