# Supplementary figures and images for: Molecular species of oxidized phospholipids in brain differentiate between learning- and memory impaired and unimpaired aged rats
Source: Amino Acids. 2022 Jul 11;54(9):1311–26. doi: 10.1007/s00726-022-03183-z (PMC9372013; doi:10.1007/s00726-022-03183-z)

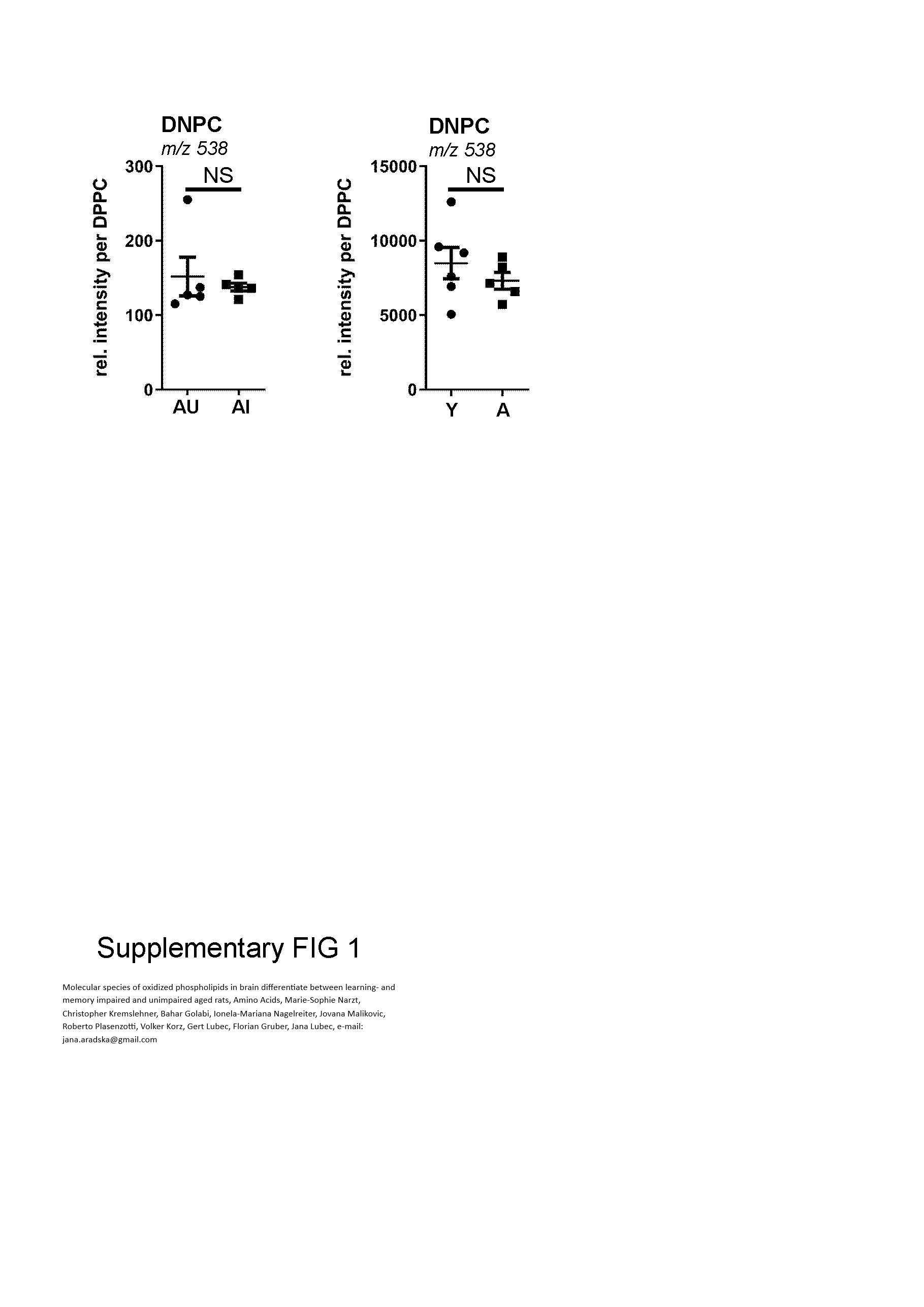

Supplement: Supplementary file 1 — Supplementary file1 (TIFF 283 KB) [file 726_2022_3183_MOESM1_ESM.tiff]

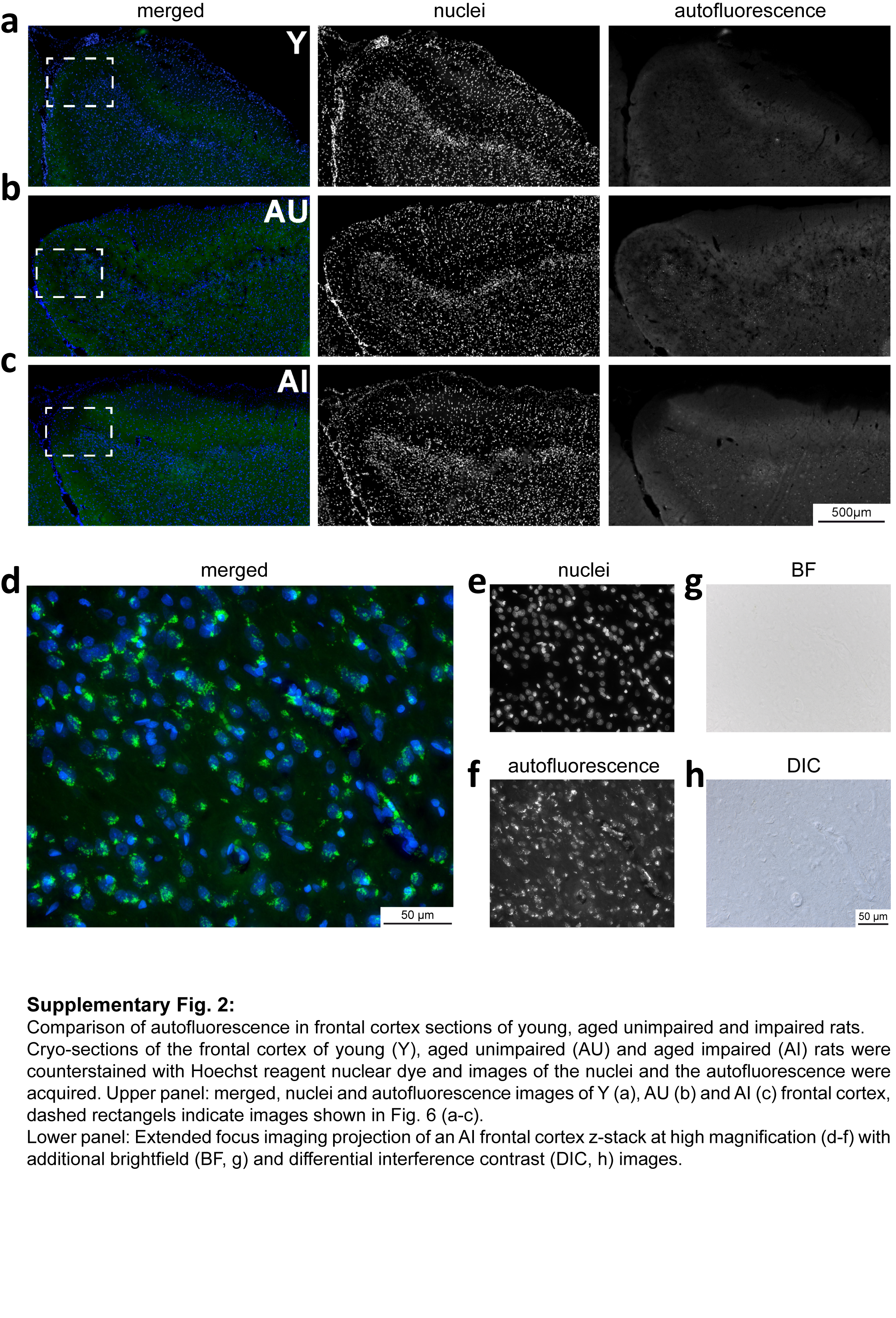

Supplement: Supplementary file 2 — Supplementary file2 (TIF 7682 KB) [file 726_2022_3183_MOESM2_ESM.tif]
